# Supplementary material for: Functional specification of CCK+ interneurons by alternative isoforms of Kv4.3 auxiliary subunits
Source: eLife. 2020 Jun 3;9:e58515. doi: 10.7554/eLife.58515 (PMC7269670; doi:10.7554/eLife.58515)
Supplement: Figure 1—source data 1. — Note that only those cells were included in this table from which all parameters could be retrieved. Thus, the number of analyzed cells may differ from specific analysis of the main text. [file elife-58515-fig1-data1.docx]

|  | **TOR**  **(n = 114)** | **RS**  **(n = 124)** | **p** | **U** | **Z** |
| --- | --- | --- | --- | --- | --- |
|  |  |  | **Mann-Whitney test** | | |
| Input resistance (MΩ) | 141.76 ± 6.86 | 138.7 ±7.01 | 0.52 | 6730.5 | -0.64 |
| **Number of initial APs @-80 mV** | **0.53 ± 0.08** | **5.09 ± 0.13** | **0** | **14077** | **13.47135** |
| **Delay of first AP @ -80 mV (ms)** | **245 ± 12.79** | **46.22 ± 2.23** | **2.3*10^-27^** | **1318** | **-10.84** |
| AP threshold @ -80 mV (mV) | -38.12 ± 0.41 | -37.96 ± 0.37 | 0.96 | 7095 | 0.05 |
| AP half width @ -80 mV (ms) | 0.49 ± 0.01 | 0.51 ± 0.01 | 0.5 | 6707.5 | -0.68 |
| AHP minimum @ -80 mV (mV) | -14.9 ± 0.29 | -14.51 ±0.3 | 0.78 | 7214.5 | 0.28 |
| AP absolute peak @ -80 mV (mV) | 37.54 ± 0.62 | 38.54 ± 0.69 | 0.24 | 7698 | 1.17 |
| AP dV/dt maximum @ -80 mV (mV/ms) | 491.6 ± 13.64 | 510.86 ± 16.88 | 0.53 | 7398.5 | 0.62 |
| **Number of initial APs @-60 mV** | **3.82 ± 0.11** | **4.74 ± 0.1** | **1.74 *10^-8^** | **9973.5** | **5.64** |
| **Delay of first AP @ -80 mV (ms)** | **75.44 ± 4.21** | **43.68 ± 2.02** | **3.31*10^-13^** | **3204** | **-7.28** |
| AP threshold @ -60 mV (mV) | -37.48 ± 0.4 | -37.65 ± 0.4 | 0.97 | 7091 | 0.04 |
| AP half width @ -60 mV (ms) | 0.49 ± 0.01 | 0.51 ± 0.01 | 0.14 | 7848 | 1.47 |
| AHP minimum @ -60 mV (mV) | -15.51 ± 0.3 | -14.36 ± 0.31 | 0.02 | 8305.5 | 2.33 |
| AP absolute peak @ -60 mV (mV) | 37.85 ± 0.61 | 39.13 ± 0.7 | 0.16 | 7815 | 1.41 |
| AP dV/dt maximum @ -60 mV (mV/ms) | 485.94 ± 13.47 | 484.02 ± 13.64 | 0.92 | 7012.5 | -0.1 |
