# Supplementary material for: Functional specification of CCK+ interneurons by alternative isoforms of Kv4.3 auxiliary subunits
Source: eLife. 2020 Jun 3;9:e58515. doi: 10.7554/eLife.58515 (PMC7269670; doi:10.7554/eLife.58515)
Supplement: Figure 4—source data 1. — Each cell (n = 10) was simulated both as TOR and RS type by exchanging ISA only. With the exception of ISA, all parameters were identical in the two conditions for individual cells. Some parameters were fixed to decrease computational demand (gray), whereas other parameters were variable between cells to optimally reproduce the average firing properties. Fixed conductance values derived from previous publications. [file elife-58515-fig4-data1.docx]

|  | **Parameter** | **TOR** | **RS** |
| --- | --- | --- | --- |
| **Passive properties** (uniform) | **cm** (µf/cm^2^) | 0.97 ± 0.03 | |
|  | **gl** (S/cm^2^) | 4.15*10^-5^ ± 4.95*10^-6^ | |
|  | **Ra** (Ωcm) | 120 (Fixed) | |
| **Soma** | **gNa** (S/cm^2^) | 0.49 ± 0.15 | |
|  | **gCa** (S/cm^2^) | 10*10^-3^ ± 1.58*10^-3^ | |
|  | **gBK** (S/cm^2^) | 10*10^-3^ ± 4.08*10^-3^ | |
|  | **BK tau** (ms) | 41.67 ± 5.27 | |
|  | **gKDR** (S/cm^2^) | 0.36 ± 0.17 | |
|  | **gKM** (S/cm^2^) | 2*10^-5^ (Fixed) | |
|  | **gH** (S/cm^2^) | 10^-4^ (Fixed) | |
|  | **gTOR** (S/cm^2^) | **44*10^-5^ ± 7*10^-5^** | **0** |
|  | **gRS** (S/cm^2^) | **128*10^-5^ ± 20.3*10^-5^** | **297*10^-5^ ± 47.2*10^-5^** |
| **Dendrite** | **gTOR** (S/cm^2^) | **14.7*10^-5^ ± 2.33*10^-5^** | **0** |
|  | **gRS** (S/cm^2^) | **42.7*10^-5^ ± 6.78*10^-5^** | **99.1*10^-5^ ± 15.7*10^-5^** |
|  | **gKDR** (S/cm^2^) | 7*10^-5^ (Fixed) | |
| **Axon Initial Segment** | **gNaax** (S/cm^2^) | 5 (Fixed) | |
|  | **gKDR** (S/cm^2^) | 1.5 (Fixed) | |
| **Axon** | **gNa** (S/cm^2^) | 0.1 (Fixed) | |
|  | **gKDR** (S/cm^2^) | 0.1 (Fixed) | |
