# Supplementary material for: Functional specification of CCK+ interneurons by alternative isoforms of Kv4.3 auxiliary subunits
Source: eLife. 2020 Jun 3;9:e58515. doi: 10.7554/eLife.58515 (PMC7269670; doi:10.7554/eLife.58515)
Supplement: Transparent reporting form [file elife-58515-transrepform.pdf]

eLIFE
Westbrook Centre, Milton Road
Cambridge CB4 1YG
UK
P 01223 855340
W elifesciences.org
T @elife

eLife's transparent reporting form

We encourage authors to provide detailed information *within their submission* to facilitate the interpretation and replication of experiments. Authors can upload supporting documentation to indicate the use of appropriate reporting guidelines for health-related research (see EQUATOR Network), life science research (see the BioSharing Information Resource), or the ARRIVE guidelines for reporting work involving animal research. Where applicable, authors should refer to any relevant reporting standards documents in this form.

Sample-size estimation

- You should state whether an appropriate sample size was computed when the study was being designed
- You should state the statistical method of sample size computation and any required assumptions
- If no explicit power analysis was used, you should describe how you decided what sample (replicate) size (number) to use

Please outline where this information can be found within the submission (e.g., sections or figure legends), or explain why this information doesn't apply to your submission:

No statistical methods were used to predetermine sample sizes, but our samples are similar to or exceed those reported in previous publications and that are generally employed in the field. The size of the samples were sufficient for the applied statistical tests.
Analysis of morphology, immunolabelling and RNA content of the recorded cells was performed blind to the electrophysiological phenotype of neurons.

Replicates

- You should report how often each experiment was performed
- You should include a definition of biological versus technical replication
- The data obtained should be provided and sufficient information should be provided to indicate the number of independent biological and/or technical replicates
- If you encountered any outliers, you should describe how these were handled
- Criteria for exclusion/inclusion of data should be clearly stated
- High-throughput sequence data should be uploaded before submission, with a private link for reviewers provided (these are available from both GEO and ArrayExpress)

Please outline where this information can be found within the submission (e.g., sections or figure legends), or explain why this information doesn't apply to your submission:

Numbers of cells included in individual analysis are clearly stated within the text.

Inclusion criteria: only those cells were included in each data set that were either immunolabelled with CCK and/or CB1 antibody or their sequencing revealed significant amount of CCK- and CB1-mRNA was detected after the recordings.
Exclusion criteria for RNAseq analysis: at least 3000 genes should be detected in individual cells. Cells where check for potential contamination from neigboring cells based on criteria described in previous publication.
Exclusion criteria for voltage-clamp recordings: maximum access resistance value without significant deterioration during recordings.
These criteria are clearly stated in the manuscript in the Results and Methods sections.

eLife Sciences Publications, Ltd is a limited liability non-profit non-stock corporation incorporated in the State of Delaware, USA, with company number 5030732, and is registered in the UK with company number FC030576 and branch number BR015634 at the address Westbrook Centre, Milton Road Cambridge CB4 1YG, UK | March 2019

Statistical reporting

- Statistical analysis methods should be described and justified
- Raw data should be presented in figures whenever informative to do so (typically when N per group is less than 10)
- For each experiment, you should identify the statistical tests used, exact values of N, definitions of center, methods of multiple test correction, and dispersion and precision measures (e.g., mean, median, SD, SEM, confidence intervals; and, for the major substantive results, a measure of effect size (e.g., Pearson's r, Cohen's d)
- Report exact p-values wherever possible alongside the summary statistics and 95% confidence intervals. These should be reported for all key questions and not only when the p-value is less than 0.05.

Please outline where this information can be found within the submission (e.g., sections or figure legends), or explain why this information doesn't apply to your submission:

Statistical analysis methods along with N, exact p and t values, and degree of freedom are indicated within the main text and/or figure legends. R(square) value is included for fits.
Individual data points or values are shown on figures for RNAseq and recording data on Figures 2, 3, 5. For other data, average data are shown together with multiple representative examples.

(For large datasets, or papers with a very large number of statistical tests, you may upload a single table file with tests, Ns, etc., with reference to sections in the manuscript.)

Group allocation

- Indicate how samples were allocated into experimental groups (in the case of clinical studies, please specify allocation to treatment method); if randomization was used, please also state if restricted randomization was applied
- Indicate if masking was used during group allocation, data collection and/or data analysis

Please outline where this information can be found within the submission (e.g., sections or figure legends), or explain why this information doesn't apply to your submission:

Not applicable for our data sets.

Additional data files ("source data")

- We encourage you to upload relevant additional data files, such as numerical data that are represented as a graph in a figure, or as a summary table
- Where provided, these should be in the most useful format, and they can be uploaded as "Source data" files linked to a main figure or table
- Include model definition files including the full list of parameters used
- Include code used for data analysis (e.g., R, MatLab)
- Avoid stating that data files are "available upon request"

Please indicate the figures or tables for which source data files have been provided:

Complete mRNA data of the included cells are uploaded to NCBI GEO database (https://www.ncbi.nlm.nih.gov/geo/) with accession number: GSE133951
